# Halogen-Driven Tunability in Cubic KZnX_3_ (X = F–I) Halide Perovskites: A First-Principles Study

**DOI:** 10.3390/ijms27062561

**Published:** 2026-03-11

**Authors:** Łukasz Szeleszczuk

**Affiliations:** Department of Organic and Physical Chemistry, Medical University of Warsaw, 1 Banacha Str., 02-097 Warsaw, Poland; lukasz.szeleszczuk@wum.edu.pl; Tel.: +48-501-255-121

**Keywords:** DFT, perovskites, elastic properties, electronic properties, optical properties

## Abstract

This paper systematically studied the structural, mechanical, electronic, and optical characteristics of cubic KZnX_3_ (X = F, Cl, Br, and I) perovskites through the density functional theory (DFT) in the Quantum Espresso framework. Structural optimization and stability analyses confirm that all compounds crystallize in the cubic Pm-3m phase and are thermodynamically, mechanically, and dynamically stable. Elastic constants indicate that the materials are anisotropic and ductile in nature. Calculations of Debye temperatures show a systematic decrease of 402 K (KZnF_3_) to 158 K (KZnI_3_), which is related to the increasing mass of halogen and its impact on the rigidity of the lattice. Electronic structure calculations show that all compounds are indirect bandgap semiconductors, with bandgaps systematically decreasing from 4.24 eV (KZnF_3_) to 0.86 eV (KZnI_3_) at the HSE06 level, enabling tunable semiconducting characteristics for optoelectronic applications. The analysis of the density of states and charge density indicates that the bonding between Zn and X is mixed ionic and covalent and that the bonding between K and X is mostly ionic. Calculations of optical properties show an increase in polarizability, absorption, refractive index and plasmonic response when heavier halogen is used, highlighting the potential of KZnX_3_ perovskites for photovoltaic and optoelectronic devices. Overall, halogen substitution in KZnX_3_ provides an effective strategy for tailoring electronic and optical properties.

## 1. Introduction

ABX_3_ cubic perovskite crystals, composed of mono- and divalent cations and a monovalent halogen anion (X), have attracted significant attention from both theoretical and experimental perspectives [1,2,3]. This has been driven by the fact that they have a comparatively straightforward crystal structure, are easy to synthesize, allow flexible doping, and comprise a wide variety of physical behavior such as electrical, optical, magnetic, piezoelectric and ferroelectric behavior [4,5,6,7]. Thanks to these characteristics, the halide perovskites have a wide range of practical uses, including a high-performance optical component, birefringence-free lenses, and electronics and magnetism functional materials. More so, they offer useful information regarding geophysical processes, especially in the lower mantle of the Earth, due to their physical characteristics [8,9,10].

Fluoride perovskites are an important subfamily of halide perovskites and have important technical applications in deep-ultraviolet (DUV) optics. Their large band gaps and high optical clarity can be utilized in DUV optoelectronics, scintillators and luminescent applications [11,12,13]. Here, KZnF_3_ has been extensively experimentally as well as theoretically investigated in regard to its structural stability and possible application as a radiation detector. Past studies of KZnF_3_ have included its synthesis, lattice dynamics, high-pressure phase behavior, elastic properties and optical response and the influences of transition-metal dopants on its crystal field and electronic structure [14,15,16,17,18,19,20,21,22].

Nevertheless, widespread focus has been placed on KZnF_3_, and other members of the KZnX_3_ (X = F, Cl, Br, and I) family have not been studied in detail, particularly on a first-principles scale. Heavier halogen analogues such as KZnCl_3_, KZnBr_3_, and KZnI_3_ lack any significant theoretical information, even though they could have some technological importance. Specifically, the change in the band gap of the series of halogen, with KZnF_3_ having a very large band gap, indicates that the replacement of bigger and less electronegative halogen atoms may cause extensive modifications in the electronic behavior, optical behavior, and thermodynamic behavior. The trends demonstrate a need to understand how to tune the material performance to more practical uses.

Out of these gaps in knowledge, the current work is a systematic and comparative first-principles investigation of the structural, electronic, elastic, optical, phononic, and thermodynamic characteristics of the complete KZnX_3_ (X F, Cl, Br, and I) family. The use of KZnF_3_ as a reference compound, although an established one, is not novel in our study, as the other halide members have not been investigated previously. We calculate the band structures, density of states, optical functions, phonon dispersions, group velocities, and the thermal parameters of these materials with respect to temperature, providing a full picture in the theoretical perspective of the physical properties of these materials.

This comprehensive study is expected to enrich the fundamental understanding of halide perovskite systems and provide a robust theoretical framework to guide future experimental investigations and potential technological applications. The remainder of this paper is organized as follows: Section 2 describes the computational methodology; Section 3 presents and discusses the structural, elastic, electronic, optical, and thermodynamic properties of KZnX_3_ compounds; and Section 4 summarizes the main conclusions of this work.

## 2. Result and Discussion

### 2.1. Structural and Thermodynamic Stability

Structural properties of the KZnX_3_ (X = F, Cl, Br, and I) compounds were studied systematically, where KZnF_3_ has been used as the reference parent perovskite compound. KZnF_3_ is cubic perovskite with space group Pm-3m (space group 221), with a formula unit per unit cell [23]. In this structure, the Zn atoms are located in the center of the cubical unit cell, the K atoms are on the corners of the unit cell and the halogen atoms are at the face-centered positions. The atom coordinates are K (0, 0, 0), Zn (0.5, 0.5, 0.5) and X (0, 0.5, 0.5), which are located at the 1a, 1b, and 3c Wyckoff sites, respectively.

**Figure 1:**
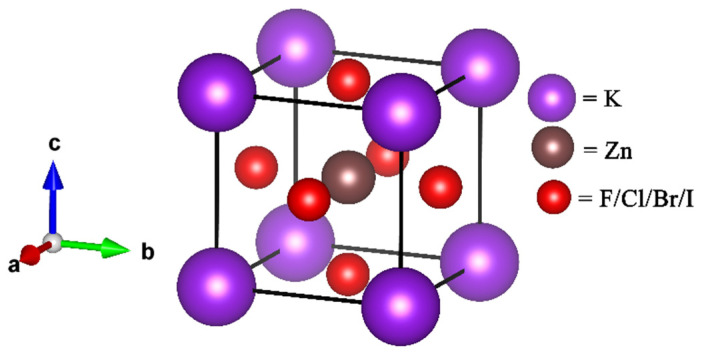
Crystal structure of KZnX_3_ compounds.

Figure 1 illustrates the unit-cell structure, while Table 1 summarizes the optimized lattice constants, unit-cell volumes, and formation energies of KZnX_3_ obtained in this work. The resulting lattice constants are consistent with experimental values as well as with other theoretical values reported in the past. As commonly observed, the GGA approach slightly overestimates or underestimates the lattice parameters; nevertheless, the overall consistency confirms the reliability of the adopted computational methodology.

All KZnX_3_ compounds (X = F, Cl, Br, and I) were investigated using the same computational framework, and their optimized structural parameters and formation energies are compiled in Table 1. All the investigated compounds have their optimum lattice constants, unit-cell volumes, and formation energies presented in Table 1. A systematic increase in lattice constants and unit-cell volumes is observed as the halogen is varied from F to Cl, Br, and I, which can be attributed to the increasing ionic radius of the halogen atoms. In contrast, the formation energies decrease progressively along the series, indicating a gradual reduction in thermodynamic stability. For KZnF_3_, the calculated structural parameters are in good agreement with the available experimental data, further validating the reliability of our computational approach. KZnF_3_, as the parent compound, is therefore used as a reference to analyze and interpret these trends.

Negative formation energies validate that all KZnX_3_ (X = F, Cl, Br, and I) compounds are thermodynamically and energetically favorable to prepare. The standard expression was used in computing the enthalpy of formation *E_f_* [26]:

(1) $$E_{f}=\frac{E_{Total}-\left(n_{K}E_{K}^{solid}+n_{Zn}E_{Zn}^{solid}+n_{X}E_{X}^{solid}\right)}{n_{K}+n_{Zn}+n_{X}}$$

where *E_Total_* is the DFT total energy of the unit cell of KZnX_3_, and $E_{K}^{solid}$, $E_{Zn}^{solid}$ and $E_{X}^{solid}$ are the energies of elemental K, Zn and halogen X in their most stable solid phases. n_K_, n_Zn_, and n_X_ denote the number of K, Zn and X atoms in the unit cell, respectively. The calculated formation energies *E_f_* are uniformly negative, indicating that the formation of the KZnX_3_ perovskite compounds from their constituent elemental solids is energetically favorable. This thermodynamic behavior suggests that KZnX_3_ compounds can be experimentally synthesized under appropriate conditions.

**Figure 2:**
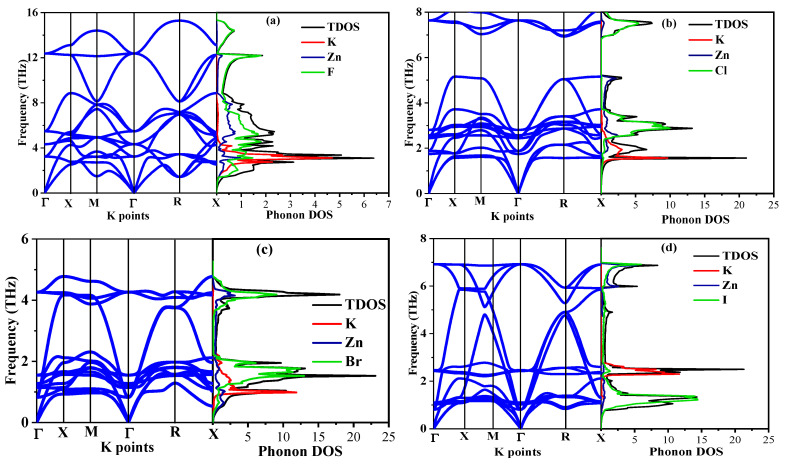
Phonon dispersion curve with phonon density of state of the (**a**) KZnF_3_, (**b**) KZnCl_3_, (**c**) KZnBr_3_ and (**d**) KZnI_3_ compounds.

Figure 2 provides the phonon dispersion relations and phonon density of states (PDOS) of the KZnX_3_ compounds. Both systems have the typical three acoustic and 12 optical phonon branches of the five atom primitive cell of the cubic perovskite structure. Notably, the existence of any imaginary phonon mode is not present anywhere within the Brillouin zone of any of the halide compositions, and therefore the dynamical stability of the cubic phase is undisputed. KZnF_3_ exhibits the highest vibrational frequencies of the series, including optical branches up to approximately 16 THz per the very strong Zn-F interactions and the low mass of fluorine. Varying the halogen atom, F to Cl to Br to I, a systematic phonon softening is evident, manifested by the lowering of the highest optical frequencies (the maximum optical frequencies of KZnCl_3_, KZnBr_3_, and KZnI_3_ being roughly 8, 10 and 8 THz, respectively), and the flattening of the optical modes. This is due to the fact that the ionic radii and atomic masses of the halogen progressively increase, which reduces the rigidity of the bonds and drags the PDOS to the lower frequencies. The PDOS analysis shows that the low-frequency acoustic region is predominantly governed by the heavier K and Zn cations, whereas the high-frequency optical modes are mainly dominated by the halogen (X) atoms. A pronounced overlap between the Zn and X vibrational states in the mid- to high-frequency regions indicates strong covalent Zn–X interactions within the ZnO_6_ octahedra. These coupled vibrations play a central role in determining the structural rigidity and lattice stability of the KZnX_3_ perovskite framework. In contrast, for KZnI_3_, the iodine atom exhibits a dominant contribution in the low-frequency region, reflecting its large atomic mass and the resulting phonon softening effects, which further influence the lattice dynamics and vibrational stability of the system.

The nearly flat phonon branches observed between the X and M high-symmetry points indicate low group velocity vibrational modes, which correspond to highly localized lattice vibrations. Physically, this flat dispersion reflects weak interatomic force constants for the corresponding vibrational modes, meaning that these phonons propagate poorly through the lattice and behave more like localized or quasi-localized vibrations rather than extended propagating waves. In perovskite structures, such flat modes are commonly associated with octahedral tilting, bending, and vibrational motions of the ZnX_6_ units, as well as vibrations involving the heavier halogen atoms, which naturally lead to reduced phonon dispersion due to their larger mass and weaker restoring forces. These low-dispersion phonon modes contribute to enhanced phonon scattering and reduced thermal transport, which is consistent with the relatively low Debye temperatures and ductile mechanical behavior observed in these compounds. Therefore, the flat region between X and M is a clear signature of localized lattice dynamics and weak phonon propagation, providing microscopic insight into the vibrational, thermal, and mechanical properties of KZnX_3_ perovskites. Altogether, the phonon spectra show that the whole KZnX_3_ family is dynamically stable and the progressive softening of F to I reflects the inherent mass- and bonding-driven vibrational response of the halide perovskites.

### 2.2. Elastic Properties

The elastic response of the materials under stress can be used to determine their mechanical stability, inability to be deformed when subjected to stress, and stiffness [27]. Elastic constants characterize the mechanical behaviors of crystalline solids in the ground state, and are of significant use in investigating the structural stability of crystalline solids. The second-order elastic constants of the cubic KZnX_3_ (X = F, Cl, Br, and I) compounds were computed with the IR-Elast package and the results are summarized in Table 2.

In the case of cubic crystals, the Born criteria of stability in mechanics are: C_11_ > 0, C_44_ > 0, C_11_ − C_12_ > 0, and B > 0 [28,29]. These conditions are met in all the calculated elastic constants of KZnX_3_, which proves that the compounds are mechanically stable. In the case of KZnF_3_, the current elastic constants are well consistent with the previously known theoretical values [22], which also proves the accuracy of our calculations. By employing more elastic parameters, the bulk modulus (*B*), shear modulus (*G*), Young’s modulus (*Y*), Poisson’s ratio (*ν*), elastic anisotropy factor (*A*) and Cauchy pressure (*Cp*) were obtained by using calculated elastic constants.

The quantities that are commonly used to describe the resistance to volume compression, opposition to plastic deformation, and stiffness of a material are the bulk modulus (*B*), shear modulus (*G*), and Young’s modulus (*Y*), respectively [7,30]. The following relation was used to evaluate these parameters using the Voigt–Reuss–Hill approximation [7,8].

(2) $$B=(C_{11}+{2C}_{12})/3$$

(3) $$G=\frac{1}{2}(G_{V}+G_{R})\;$$

(4) $$G_{V}=\frac{1}{5}(C_{11}-C_{12}+{3C}_{44})\;$$

(5) $$G_{R}=\frac{{5C}_{44}(C_{11}-C_{12})}{{4C}_{44}+3(C_{11}-C_{12})}$$

(6) $$Y=\frac{9BG}{3B+G}$$

*B*, *G* and *Y* are calculated to obtain the values, as indicated in Table 3. The order is observed to decline systematically as KZnF_3_ > KZnCl_3_ > KZnBr_3_ > KZnI_3_, meaning that KZnF_3_ is the most stiff and the least compressible, whereas KZnI_3_ is relatively softer and more compressible. This is due to the fact that the ionic radius of the halogen atom is increasing and there is a corresponding decrease in the strength of interatomic bonding between F and I.

The Cauchy pressure Cp = C_12_ − C_44_, Pugh ratio *B*/*G* and Poisson ratio (*ν*) were used to analyze the ductile or brittle nature of the materials. When the Cauchy pressure value is positive (negative), it shows ductile (brittle) behavior. Moreover, the Poisson ratio, which is more than 0.26, and the Pugh ratio, which is more than 1.75, are typical of ductile materials [31,32,33,34]. Table 3 illustrates that all KZnX_3_ compounds are ductile, all of them have positive Cauchy pressure, the Poisson ratio value is more than 0.26, and the *B*/*G* ratio value is more than 1.75. KZnI_3_ has the best values of *B*/*G* and the Poisson ratio, which means that it has a relatively high ductility. The Zener anisotropy factor (A) was used to determine the elastic anisotropy that greatly determines the formation of cracks and mechanical reliability [11]:

(7) $$A=\frac{{2C}_{44}}{{3C}_{11}-C_{12}}$$

An elastic material that is isotropic has A = 1, and non-unity values denote elastic anisotropy. The results of the calculations of the anisotropy factors of all the KZnX_3_ shows that they are not equal to one (Table 3), which shows that the compounds have anisotropic elastic behavior.

### 2.3. Debye Temperature

The Debye temperature (*θ_D_*) is one of the key physical quantities, which characterizes the vibrational properties of the lattice and determine the temperature dependence of the heat capacity of solids. Its value gives valuable information on the interatomic bonding strength, rigidity of the lattice and thermal behavior. Several theoretical approaches have been developed to estimate (*θ_D_*), among which mechanical models based on elastic constants are widely employed due to their reliability and simplicity. In the present system, (*θ_D_*) is evaluated using the classical relation. In this system, the determination of *θ_D_* is possible with the help of the classical relation [35]:

(8) $$\Theta_{D}=\frac{h}{k_{B}}[(\frac{3n}{4\pi })N_{A}\rho /M]V_{m}$$

with *h* being the constant of Planck, *k_B_* being the constant of Boltzmann, *n* being the number of atoms per unit formula, *N_A_* being the number of Avogadro, rho being the density, *M* being the molecular mass per unit formula and *V_m_* being the average velocity of sound. The longitudinal and transverse sound velocities are used to get the average sound velocity as [36]:

(9) Vm=[13(1vl3+2vt3)]−13

The bulk modulus (*B*) and shear modulus (*G*) are used to obtain the longitudinal (*V_l_*) and transverse (*V_t_*) wave velocities by the following equations [37]:

(10) Vl=[(3B+4G)/3ρ]12

(11) and Vt=[G/ρ]12

According to Table 4, KZnF_3_ has the highest longitudinal (2655.58 ms^−1^), transverse (1459.31 ms^−1^), and average (1626.69 ms^−1^) sound velocities, making its Debye temperature the largest (401.76 K). This number is well consistent with the theoretical and experimental values of the Debye temperature already found in the literature, proving the consistency of the current calculations.

A systematic decrease in *V_l_*, *V_t_*, and *V_m_* is observed from KZnF_3_ to KZnI_3_, leading to a corresponding reduction in *θ_D_* from 401.76 K to 157.52 K. This trend can be attributed to the increasing atomic mass of the halogen element, which weakens the lattice stiffness and lowers the characteristic vibrational frequencies.

Other significant values of the Debye model can be obtained by *θ_D_*, including the Debye temperature of acoustic phonons [38,39] and the Debye frequency, which is the highest frequency of vibrational movement of atoms in thermal excitation [40,41]. The sound velocities and Debye temperature were calculated and are summarized in Table 4. As far as we know, there is no theoretical or experimental information about the Debye temperatures of KZnCl_3_, KZnBr_3_, and KZnI_3_. Thus, the current findings give practical predictions that can be considered as reference data in experimental and theoretical studies in the future.

**Table 4:** Calculated density (*ρ*), longitudinal (*V_l_*), transverse (*V_t_*), and average (*V_m_*) sound velocities, Debye temperature (*θ_D_*), and melting temperature (*T_m_*) of the KZnX_3_ (X = F, Cl, Br, and I) compounds. Available theoretical and experimental values are included for comparison.

Compounds	*ρ* (kg/m^3^)	*V_l_* (m/s)	*V_t_* (m/s)	*V_m_* (m/s)	*θ_D_* (K)	*T_m_* (K)	Ref.
KZnF_3_	3814.08	2655.58	1459.31	1626.69	401.76	1067.86	This study
	3752	5110	2811	3134	384.2	-	[25]
	4024^exp^	-	-	-	437	-	[42]
KZnCl_3_	2823.71	2530.15	1421.00	1581.27	323.58	1049.41	This study
KZnBr_3_	3843.03	1679.03	917.84	1023.52	196.91	799.73	This study
KZnI_3_	4142.88	1557.12	799.40	895.33	157.52	802.58	This study

exp—experimentally obtained value.

### 2.4. Electronic Properties

**Figure 3:**
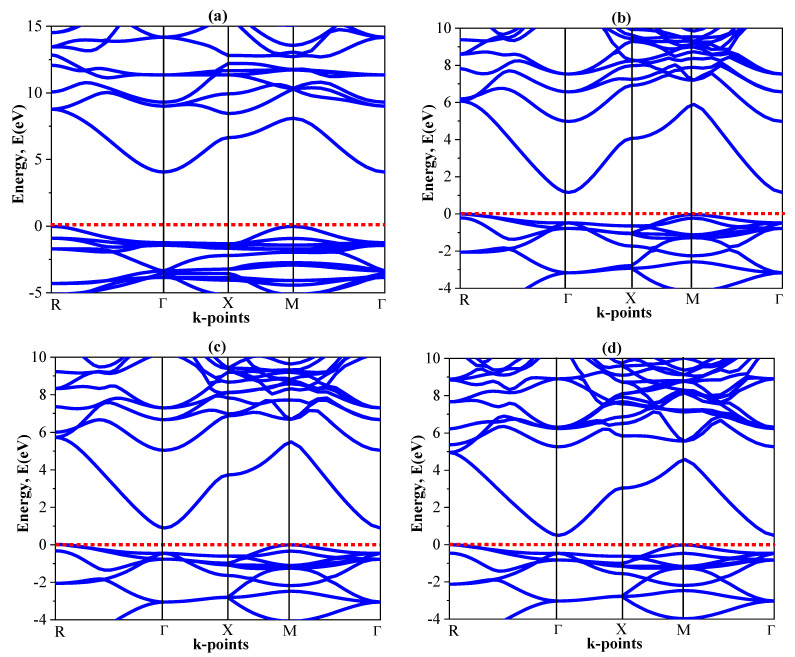
The calculated HSE06 band structure of the (**a**) KZnF_3_, (**b**) KZnCl_3_, (**c**) KZnBr_3_ and (**d**) KZnI_3_ compounds. Red dashed lines represent the cutoff 0 eV value.

In this section, we explore the electronic characteristics of KZnF_3_ in terms of the calculated energy band structures, density of states, and charge distribution. Figure 3 shows the electronic band structures of the cubic KZnX_3_ (X = F, Cl, Br, and I) along high-symmetry directions in the Brillouin zone, calculated using either the GGA-PBE or HSE06 exchange-correlation functional. The zero of energy is defined as the valence band maximum (VBM). The HSE06 band gap values for all investigated compounds are summarized in Table 5.

In the case of KZnF_3_, the VBM is at the R point, and the conduction band minimum (CBM) is at the Γ point, which is an indirect R-G band gap. The band gap of KZnF_3_ has been calculated as 3.902 eV in GGA-PBE and is found to be 4.241 eV in HSE06 functional. Figure 3a indicates that the HSE06 band structure has a distinct and broad band gap indicating the indirect wide-band-gap characteristic of the insulator/semiconductor KZnF_3_. Our predicted values are in agreement with previously reported theoretical and experimental values of the band gap of KZnF_3_, which was reported as listed in Table 5. Indeed, the HSE06 formalism does produce systematically larger band gaps than GGA-PBE, as its treatment of exchange interactions is better.

In the rest of the halide compounds KZnCl_3_, KZnBr_3_, and KZnI_3_, the values of the valence band maximum (VBM) and conduction band minimum (CBM) are observed to be at other k-points, and hence, at other k-points are indirectly semiconducting. The indirect band gap in KZnCl_3_ [Figure 3b] is much smaller than in KZnF_3_, which is calculated to be 1.626 eV (GGA-PBE) and 2.257 eV (HSE06). An additional reduction in the band gap is seen in KZnBr_3_ [Figure 3c], where the band gap reduces to 0.610 eV (GGA-PBE) and 1.111 eV (HSE06). Lastly, KZnI_3_ [Figure 3d] is the narrowest gap indirect semiconductor with GGA-PBE and HSE06 indirect band gap values of 0.362 eV and 0.860 eV, respectively. As far as we know, there is no prior theoretical and experimental band gap data in the literature on KZnCl_3_, KZnBr_3_ and KZnI_3_ that can be used as a reference in other works. The current results have been a valuable source of information for future research.

**Figure 4:**
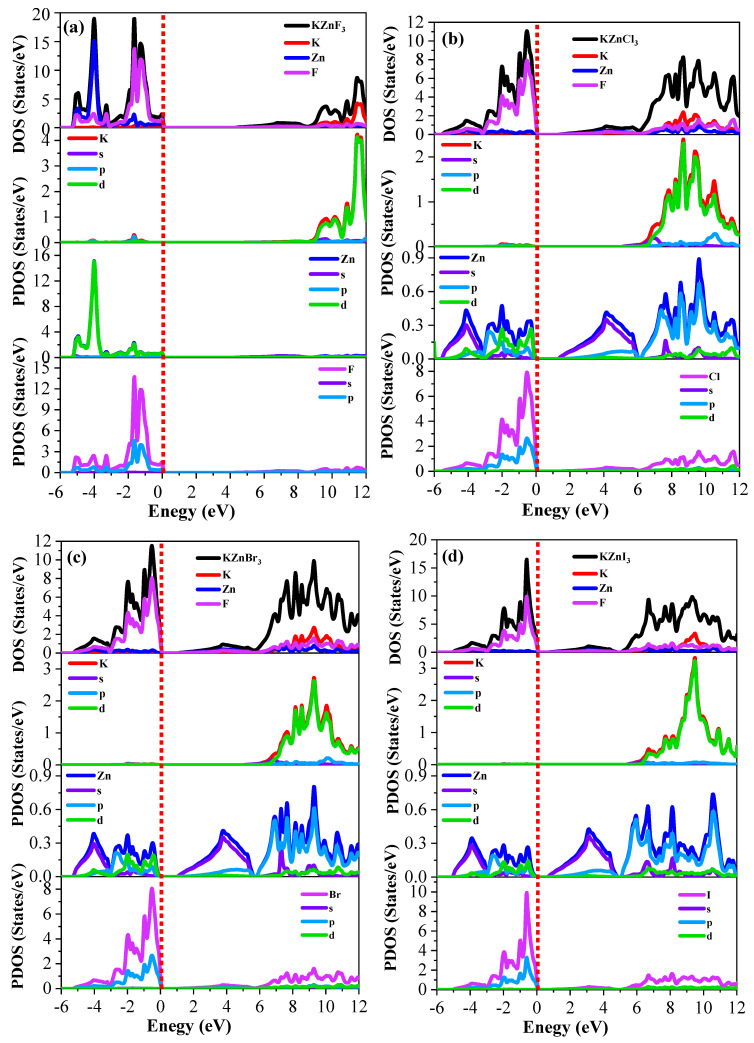
The calculated HSE06 total and partial density of states for the (**a**) KZnF_3_, (**b**) KZnCl_3_, (**c**) KZnBr_3_ and (**d**) KZnI_3_ compounds. Red dashed lines represent the cutoff 0 eV value.

In order to better understand the electronic structure, the total and partial density of states (TDOS and PDOS) were studied in the HSE06 approximation (Figure 4). The presence of halogen p states with substantial contributions of Zn-*d* states in all compounds implies intense p-d orbital hybridization at all their valence bands below the Fermi level (E = 0 eV). The contribution of the K states is mainly negligible around the Fermi level, which proves the ionic nature of K and its insignificant role in the electronic transportation. The valence band of KZnF_3_ comprises predominantly F-*p* with a Zn-*d* contribution, but the conduction band is predominantly formed with Zn-*s* and Zn-*d* contributions, as expected of a wide-band-gap insulator. In the case of KZnCl_3_, the Cl-*p* states become closer to the Fermi level, pushing the broadening of the valence band and narrowing the band gap. This is more dominant at KZnBr_3_, where Br-*p* states dominate over Zn-*d* states, as it further reduces the band gap. KZnI_3_ has I-*p* states at the lowest point of the valence band nearest to the Fermi level, so the band gap in the series is the smallest.

**Figure 5:**
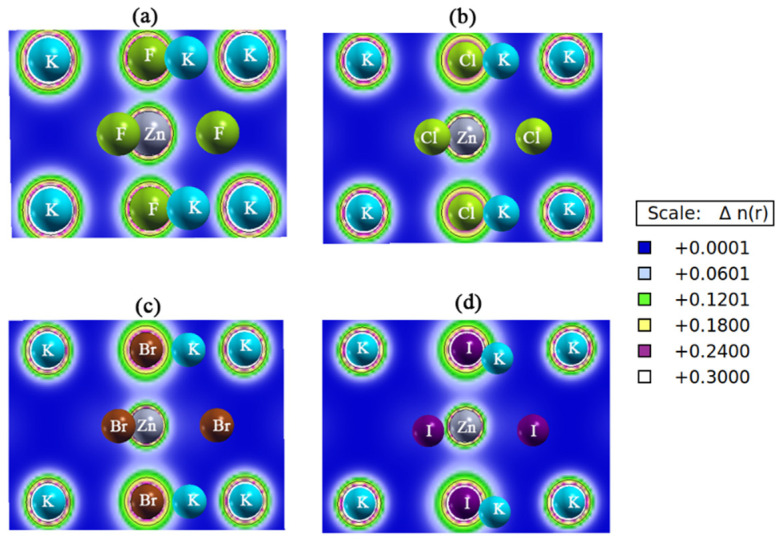
The 2D charge density plots of the (**a**) KZnF_3_, (**b**) KZnCl_3_, (**c**) KZnBr_3_ and (**d**) KZnI_3_ compounds.

The bonding behavior can be further explained by charge density analysis (Figure 5). In the case of KZnF_3_, Zn displays a directional charge density to F atoms, which implies that Zn and F atoms are partly covalently bonded, and K displays an almost spherical charge distribution, which confirms the existence of ionic K-F interactions. The same behavior is observed in KZnCl_3_, which exhibits a little lower Zn-Cl covalency, but in KZnBr_3_, the Zn-Br charge density is further delocalized, and hence weaker covalency is shown. This is the most delocalized in KZnI_3_, showing that the covalency is not strong at all. In all the halides, the K-X bonds are mostly ionic. Therefore, X-Zn bonds have a mixed ionic–covalent nature that gradually decreases from F to I, and KX bonds are mostly ionic.

The tunable electronic structure and bonding characteristics of the KZnX_3_ series enable their optoelectronic properties to be tailored for photovoltaic applications. KZnF_3_, characterized by predominantly ionic K–F interactions and a wide band gap, is well suited for use as a transparent conducting layer or electron transport material. KZnCl_3_ and KZnBr_3_, with higher Zn-X covalency and *p*-*d* hybridization, are good candidates as active absorbers of visible and near-infrared light, respectively. The delocalized KZnI_3_ has delocalized Zn-I states, allowing it to absorb lower-energy photons, which is beneficial to tandem or broad-spectrum solar cell applications. The KZnX_3_-based halogenation is a viable approach to design the electronic structure and optimize the materials towards high-performance optoelectronic and solar energy devices.

### 2.5. Optical Properties

The optical behavior of materials is determined by their electronic structure and interaction with incident photons and gives important information about their appropriation in optoelectronics [43]. The optical performances of cubic KZnX_3_ (X = F, Cl, Br, and I) compounds are organized and studied in the photon energy of 0–15 eV. The complex dielectric function is also used to obtain the frequency-dependent optical parameters.

(12) $$\varepsilon (\omega )=\varepsilon_{1}(\omega )+i\varepsilon_{2}(\omega )$$

where *ε*_1_(*ω*) and *ε*_2_(*ω*) denote the real and imaginary components, respectively.

#### 2.5.1. Dielectric Function

**Figure 6:**
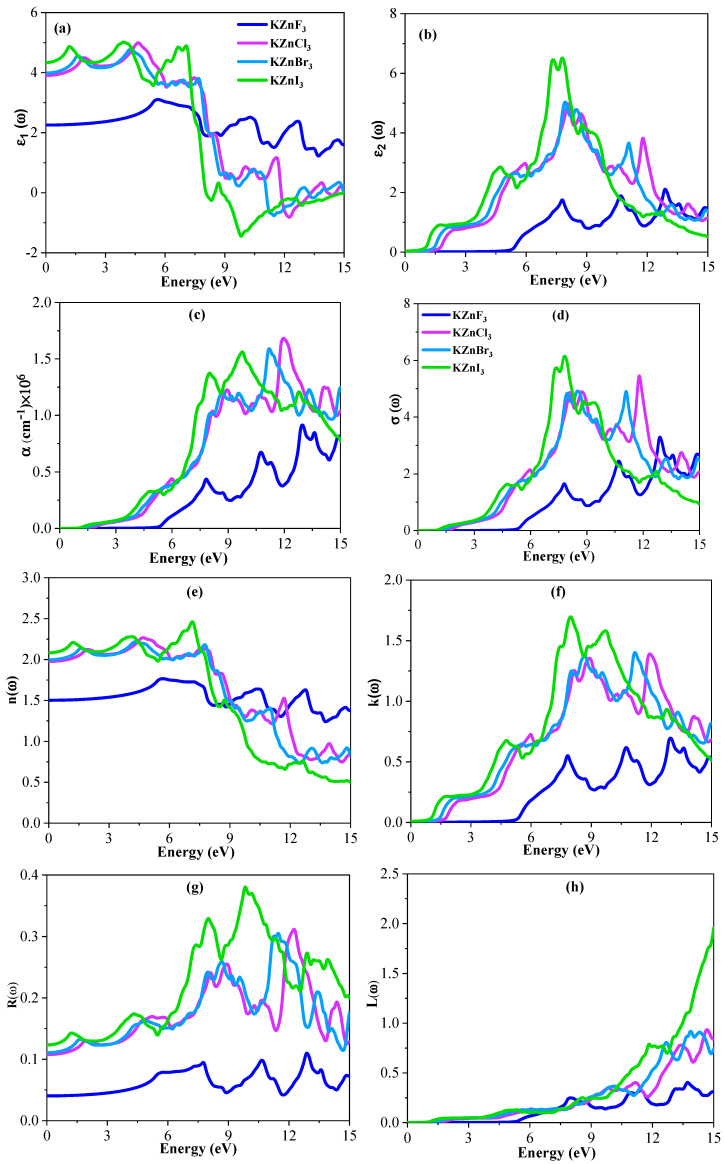
Calculated optical parameters. (**a**) Real dielectric part of dielectric constant, (**b**) imaginary dielectric part of dielectric constant, (**c**) absorption coefficient, (**d**) conductivity, (**e**) refractive index, (**f**) extinction coefficient, (**g**) reflectivity, and (**h**) loss function of the KZnX_3_ (X = F, Cl, Br, and I) compounds.

The polarization response of the material is described by the real part of the dielectric function, *ε*_1_(*ω*). As shown in Figure 6a, the static dielectric constant ε_1_(0) is lowest for KZnF_3_ and increases progressively for KZnCl_3_, KZnBr_3_, and KZnI_3_, indicating enhanced polarizability with increasing halogen atomic size. As the photon energy increases, *ε*_1_(*ω*) increases and peaks of about 3.11 at 5.62 eV for KZnF_3_, 5.02 at 4.63 eV for KZnCl_3_, 4.82 at 4.22 eV for KZnBr_3_, and 5.05 at 3.91 eV for KZnI_3_ are found, due to the strong interband electronic transitions. At stronger energies, *ε*_1_(*ω*) is smaller and oscillatory. Interestingly, at the energies of 9–12 eV, ε_1_(ω) has a negative value at KZnCl_3_, KZnBr_3_, and KZnI_3_, suggesting a plasma-like response, but KZnF_3_ is largely positive.

The imaginary component, *ε*_2_(*ω*), is the optical absorption caused by interband transitions, and it is presented in Figure 6b. The optical band gap of the compounds is reflected on the absorption onset. KZnF_3_ has a higher threshold energy of approximately 5.10 eV, which is in line with its broader band gap, with KZnCl_3_, KZnBr_3_ and KZnI_3_ having lower threshold energies of approximately 0.5–1.5 eV, which is the narrowing of the band gap as the halogen atomic number increases. Peaks of ε_2_(ω) are pronounced at 12.91 eV (2.1), 8.09 eV (4.97), 7.93 eV (5.03), and 7.33 eV (6.45) of KZnF_3_, KZnCl_3_, KZnBr_3_ and KZnI_3_, respectively. The series at KZnI_3_ has the highest intensity of absorption, which is a sign of greater absorption of ultraviolet light.

#### 2.5.2. Absorption Coefficient

Figure 6c shows the optical absorption coefficient, *α*(*ω*), versus the photon energy. All the compounds display a low photon energy and, in this regime, there is no absorption, which implies that the material is optically transparent. The absorption edge moves systematically to the lower energies of KZnF_3_ to KZnI_3_ in a progressive narrowing of the band gap as the atomic number of the halogen atom increases. The latest absorption onset and weakest absorption intensity of KZnF_3_ is observed with the highest values below a specific band gap of approximately 0.9 × 10^6^ cm^−1^. By contrast, KZnCl_3_ and KZnBr_3_ have lower absorption edges and the largest absorption coefficients, at 1.68 × 10^6^ cm^−1^ and 1.58 × 10^6^ cm^−1^, respectively, in the ultraviolet. KZnI_3_ exhibits a lower maximum absorption of about 1.55 × 10^6^ cm^−1^ and it has a stronger absorption at lower photon energies. As the photon energy increases, *α*(*ω*) increases rapidly due to intense interband electronic transitions. At higher photon energies, the absorption coefficient displays oscillatory behavior, originating from complex high-energy electronic transitions. For KZnF_3_, the optical absorption starts at a slightly higher energy than the HSE06 bandgap because the first electronic transition has a low probability. Stronger, dipole-allowed transitions appear at higher photon energies, which is why the absorption edge is shifted. Since all KZnX_3_ compounds have cubic symmetry (Pm-3m), their optical response is isotropic, so no polarization dependence is expected.

#### 2.5.3. Optical Conductivity

Figure 6d gives the photon-energy-dependent optical conductivity *σ*(*ω*). At low photon energies (0–3 eV), all the compounds exhibit negligible conductivity, showing low electronic transition. As the photon energy increases, *σ*(*ω*) rises rapidly due to the interband transitions and goes almost in step with the pattern of the absorption spectra. The highest conductivity peak for KZnF_3_ is 3.28 at 12.88 eV and 5.46 at 11.79 eV for KZnCl_3_, 4.91 at 8.50 eV for KZnBr_3_, and 6.14 at 7.82 eV for KZnI_3_. KZnI_3_ has the greatest conductivity peak at lower energies, but KZnF_3_ has the lowest conductivity peak at higher energies. Above these maxima, oscillations are produced by high-energy electronic transitions.

#### 2.5.4. Refractive Index

The essential optical parameter is the refractive index that describes the dispersion and uptake of photons that encounter the material. Past studies have shown that denser materials are likely to have higher values of refractive index [44]. The value of the refractive index against the photon energy of the KZnX_3_ compounds under study is shown in Figure 6e. The trends of the dielectric function *ε*(*ω*) are closely reflected in the refractive index spectra, thus displaying the close connection between the two optical characteristics. At the static limit (n(0)), KZnF_3_, KZnCl_3_, KZnBr_3_ and KZnI_3_ are found to have values of the refractive index of 1.50, 2.00, 1.96 and 2.08, respectively. *n*(*ω*) increases slowly with the photon energy, with the highest values of 1.77 at 5.61 eV (KZnF_3_), 2.25 at 4.64 eV (KZnCl_3_), 2.22 at 4.26 eV (KZnBr_3_), and 2.47 at 7.13 eV (KZnI_3_). It is known that these maxima are due to strong interband electronic transitions and the maxima are well added to the sharp features of the dielectric function.

#### 2.5.5. Extinction Coefficient

An optical response that is evidently halogen-dependent with increasing photon energy is observed in the extinction coefficient *k*(*ω*). KZnF_3_ has the lowest absorption and *k*(*ω*) remains low throughout most of the energy range, representing a broader band gap. KZnCl_3_ and KZnBr_3_, on the other hand, show a moderate absorption behavior, with a slow onset and strong peaks in the middle of the energy range. KZnI_3_ exhibits the greatest absorption of all the compounds, an earlier onset, and larger peak intensities associated with better optical transitions because of heavier halogen ions.

#### 2.5.6. Reflectivity

The reflectivity *R*(*ω*), which characterizes a material’s ability to reflect incident electromagnetic radiation, is presented in Figure 6g. At the static limit, the reflectivity of KZnF_3_, KZnCl_3_, KZnBr_3_, and KZnI_3_ are approximately 0.04, 0.10, 0.11, and 0.12, respectively, which decrease in order of the atomic number of halogens. The highest reflectivity is approximately 0.11 at 11.89 eV in KZnF_3_, 0.31 at 12.24 eV in KZnCl_3_, 0.30 at 11.47 eV in KZnBr_3_ and 0.38 at 9.85 eV in KZnI_3_, which implies that the optical response of iodide-based compounds is stronger.

#### 2.5.7. Energy-Loss Function

The energy-loss *L*(*ω*) is a description of the energy loss of fast electrons passing through the material. The *L*(*ω*) for all compounds is almost zero at low photon energy and tends to increase considerably at high photon energy. KZnF_3_ has the lowest maximum loss (0.41), with KZnCl_3_ and KZnBr_3_ having intermediate peaks of 0.92 and 0.93, respectively. The maximum energy-loss peak of KZnI_3_ is the highest, and it peaks strongly at approximately 1.9 around 15 eV, which means that heavier halogen ions have more plasmonic excitations.

## 3. Methods and Materials

The calculation in the present research was carried out in the context of density functional theory (DFT) by the Quantum Espresso (QE) package [45]. Norm-conserving/ultrasoft PAW [46] pseudopotentials were taken from a well-established and validated pseudopotential library (e.g., the PSLibrary provided with Quantum ESPRESSO), with explicit treatment of the valence electrons for K (3s^2^3p^6^4s^1^), Zn (3d^10^4s^2^), and halogen atoms X = F, Cl, Br, and I (ns^2^np^5^) ensuring computational reproducibility, reliability, and accuracy of the results for KZnX_3_ halide perovskites. The Generalized Gradient Approximation (GGA) in the Perdew–Burke–Ernzerhof functional (PBE) form of the exchange–correlation functional was used with ultrasoft pseudopotentials for structural optimization, mechanical analysis, and the evaluation of electronic and thermal properties [47]. Convergence tests indicated that a 60 Ry plane-wave cutoff energy and 10 × 10 × 8 Monkhorst–Pack k-point grid give well converged results, with the overall energies being 10^−6^ Ry. To obtain a more accurate description of the exchange–correlation effects, particularly for electronic and optical properties, the hybrid Heyd–Scuseria–Ernzerhof functional (HSE06) was also applied using norm-conserving pseudopotentials [48]. The energy strain method of the thermos-pw package [49,50] was used to estimate the elastic constants. The thermodynamic and dynamical stability of KZnX_3_ were studied by the finite-displacement method of the Phonopy code [51,52]. Further, the frequency of phonons, matrices of the force constant and fine-scale vibrational features were calculated with density functional perturbation theory (DFPT) [53]. For these phonon calculations, a 2 × 2 × 2 supercell was constructed, and a reduced k-point grid of 4 × 4 × 4 together with a plane-wave cutoff energy of 40 Ry was employed to ensure computational efficiency while maintaining sufficient accuracy for lattice dynamical properties. In addition, the frequency-dependent optical properties, including the dielectric function, absorption coefficient, and optical conductivity, were calculated within the independent-particle approximation using the Kubo–Greenwood formalism as implemented in Quantum ESPRESSO.

## 4. Conclusions

In this study, a comprehensive first-principles investigation of the structural, mechanical, electronic, thermal, and optical properties of cubic perovskite KZnX_3_ (X = F, Cl, Br, and I) compounds has been carried out using density functional theory. These materials are of considerable interest due to their tunable physical properties and potential applicability in future optoelectronic and energy-related technologies, highlighting the importance of reliable theoretical predictions to guide experimental efforts. The structural analysis indicates that all the KZnX_3_ compounds crystallize in the cubic Pm-3m perovskite structure and have negative formation energy, and thus they are thermodynamically stable. The computed elastic constants and mechanical parameters obtained show that all compositions pass the Born stability criteria. Ductility indicators, including the Pugh ratio, Cauchy pressure, and Poisson’s ratio, consistently reveal a ductile nature across the series, with KZnI_3_ exhibiting the highest ductility. Furthermore, deviations of the Zener anisotropy factor from unity indicate elastic anisotropy in all compounds. KZnF_3_ has the highest Debye temperature among the analyzed materials, and these are the first reported Debye temperatures of KZnCl_3_, KZnBr_3_, and KZnI_3_. Electronic band structure calculations of all KZnX_3_ compounds show them to be indirect-band-gap semiconductors. The density of states and charge density studies have shown that the X-*p* and Zn-*d* states are the main contributors to the valence bands, and the Zn state is the main contributor to the conduction bands, together with potassium, which also makes a minimal contribution near the Fermi level, although, as it is known, its role is mainly ionic in nature. The Zn–X bonds exhibit a mixed ionic–covalent character, being strongest for F and weakest for I. Optical property analysis reveals a systematic enhancement in optical response from KZnF_3_ to KZnI_3_, manifested by an increasing dielectric constant, refractive index, absorption coefficient, and optical conductivity. While KZnF_3_ remains transparent in the visible region, KZnCl_3_, KZnBr_3_, and KZnI_3_ exhibit strong absorption at lower photon energies, making them promising candidates for optoelectronic and photovoltaic applications. It should be noted that in perovskites containing heavier halogens such as Br and I, relativistic effects, particularly spin–orbit coupling (SOC), can influence the electronic band structure and modify the band gap. Although SOC was not included in the present calculations, its consideration in future studies may provide a more accurate description of the electronic properties. Additionally, the use of heavier halogens may raise potential environmental and toxicity concerns; therefore, further studies on environmental safety and material stability are desirable for practical applications.

Overall, the results presented in this work provide valuable theoretical insight and are expected to stimulate further experimental investigations of KZnX_3_ perovskite materials.

## Figures and Tables

**Table 1:** Optimized lattice constants *a*_0_ (Å), the unit-cell volume (V), and formation energy (*E*_***f***_) (eV/atom) of the KZnX_3_ (X = F, Cl, Br, and I) compounds.

Compounds	*a* (Å)	*V* (Å^3^)	*E_f_*	Ref.
KZnF_3_	4.14, 4.054^exp^, 4.06^exp^, 4.129	66.30	−2.812	[13,23,24,25]
	4.12	70.29	−2.758	This study
KZnCl_3_	4.98	123.57	−2.027	This study
KZnBr_3_	5.29	148.70	−1.955	This study
KZnI_3_	5.76	194.44	−1.795	This study

exp—experimentally obtained value.

**Table 2:** The calculated elastic constants (*C*_11_, *C*_12_, and *C*_44_) of the KZnX_3_ (X = F, Cl, Br, and I) compounds.

Compounds	*C*_11_	*C*_12_	*C*_44_	Ref.
KZnF_3_	87.11	52.86	49.81	This study
	99.20	38.00	29.10	[25]
	134.80	41.10	41.10	[22]
	111.80	49.80	31.40	[13]
KZnCl_3_	83.99	21.06	18.45	This study
KZnBr_3_	41.74	18.24	13.83	This study
KZnI_3_	42.23	17.98	9.68	This study

**Table 3:** The calculated elastic modulus (*B*, *Y*, and *G*), the Pugh ratio (*B*/*G*), Poisson’s ratio (*ν*), Cauchy pressure’s (Cp) and the Zener anisotropy factor (*A*) of the KZnX_3_ (X = F, Cl, Br, and I) compounds.

Compounds	B	*G*	*Y*	B/G	ν	Cp	A	Ref.
KZnF_3_	64.27	32.49	83.42	1.98	0.28	3.05	2.91	This study
	58.43	29.70	78.13		0.28			[3]
	70.43	31.20	81.61		0.31			[5]
KZnCl_3_	42.03	22.88	58.11	1.84	0.27	2.61	0.59	This study
KZnBr_3_	26.07	12.95	33.34	2.01	0.29	4.41	1.18	This study
KZnI_3_	26.06	10.59	27.99	2.46	0.32	8.30	0.80	This study

**Table 5:** Calculated bandgap values of the KZnX_3_ (X = F, Cl, Br, and I) compounds.

Compounds	KZnF_3_	KZnCl_3_	KZnBr_3_	KZnI_3_
GGA PBE	3.902 ^This study^ Experimental: 3.700, 3.664, 3.680 [13,22,25]	1.626	0.610	0.362
HSE06	4.241	2.257	1.111	0.860
Nature	Indirect	Indirect	Indirect	Indirect

## Data Availability

The original contributions presented in this study are included in the article. Further inquiries can be directed to the corresponding author.

## References

[1] Akkerman Q.A., D’Innocenzo V., Accornero S., Scarpellini A., Petrozza A., Prato M., Manna L. (2015). Tuning the Optical Properties of Cesium Lead Halide Perovskite Nanocrystals by Anion Exchange Reactions. J. Am. Chem. Soc..

[2] Akkerman Q.A., Gandini M., Di Stasio F., Rastogi P., Palazon F., Bertoni G., Ball J.M., Prato M., Petrozza A., Manna L. (2016). Strongly Emissive Perovskite Nanocrystal Inks for High-Voltage Solar Cells. Nat. Energy.

[3] Adewale A.A., Chik A., Adam T., Yusuff O.K., Ayinde S.A., Sanusi Y.K. (2021). First Principles Calculations of Structural, Electronic, Mechanical and Thermoelectric Properties of Cubic ATiO_3_ (A= Be, Mg, Ca, Sr and Ba) Perovskite Oxide. Comput. Condens. Matter.

[4] Navrotsky A., Weidner D.J. (1989). Perovskite: A Structure of Great Interest to Geophysics and Materials Science. Geophys. Monogr. Ser..

[5] Hossain A., Akhtaruzzaman M., Uddin M.M. (2026). DFT-Based Design of X_3_BiY_3_ (X = Mg, Sr, Ba; Y = F, Cl) Perovskites: From Wide-Gap Insulators to Promising Semiconductors for Solar Cell Applications. J. Phys. Chem. Solids.

[6] Absike H., Baaalla N., Attou L., Labrim H., Hartiti B., Ez-zahraouy H. (2022). Theoretical Investigations of Structural, Electronic, Optical and Thermoelectric Properties of Oxide Halide Perovskite ACoO_3_ (A = Nd, Pr or La). Solid State Commun..

[7] Ghebouli B., Ghebouli M.A., Bouhemadou A., Fatmi M., Khenata R., Rached D., Ouahrani T., Bin-Omran S. (2012). Theoretical Prediction of the Structural, Elastic, Electronic, Optical and Thermal Properties of the Cubic Perovskites CsXF_3_ (X = Ca, Sr and Hg) under Pressure Effect. Solid State Sci..

[8] O’Keeffe M., Bovin J.-O. (1979). Solid Electrolyte Behavior of NaMgF_3_: Geophysical Implications. Science.

[9] Street J.N., Wood I.G., Knight K.S., Price G.D. (1997). The Influence of Thermal Vibrations on the Average Structure of Cubic Perovskite: A Combined Molecular Dynamics and Neutron Diffraction Study. J. Phys. Condens. Matter.

[10] Hossain A., AlMohamadi H., Wang B., Akhtaruzzaman M., Uddin M.M. (2025). Structural, Electronic, Optical, Mechanical, and Thermal Properties of A_3_MCl_3_ (A = Mg, Ca; M = N, Bi) Halide Perovskites: A First-Principles Study. Comput. Condens. Matter.

[11] Yamanoi K., Nishi R., Takeda K., Shinzato Y., Tsuboi M., Luong M.V., Nakazato T., Shimizu T., Sarukura N., Cadatal-Raduban M. (2014). Perovskite Fluoride Crystals as Light Emitting Materials in Vacuum Ultraviolet Region. Opt. Mater..

[12] Lee J., Shin H., Lee J., Chung H., Zhang Q., Saito F. (2003). Mechanochemical Syntheses of Perovskite KMIIF_3_ with Cubic Structure (MII = Mg, Ca, Mn, Fe, Co, Ni, and Zn). Mater. Trans..

[13] Meziani A., Heciri D., Belkhir H. (2011). Structural, Electronic, Elastic and Optical Properties of Fluoro-Perovskite KZnF_3_. Phys. B Condens. Matter.

[14] Young E.F., Perry C.H. (1967). Infrared Studies of Some Perovskite Fluorides. II. Multiphonon Spectra. J. Appl. Phys..

[15] Rousseau M., Gesland J.Y., Hennion B., Heger G., Renker B. (1981). Low Energy Phonon Dispersion Curves of KZnF3 and CsCaF3. Solid State Commun..

[16] Lehner N., Rauh H., Strobel K., Geick R., Heger G., Bouillot J., Renker B., Rousseau M., Stirling W.G. (1982). Lattice Dynamics, Lattice Instabilities and Phase Transitions in Fluoride Perovskites. J. Phys. C Solid. State Phys..

[17] Burriel R., Bartolome J., Gonzalez D., Navarro R., Ridou C., Rousseau M., Bulou A. (1987). KZnF_3_ Cubic Perovskite. Heat Capacity and Lattice Dynamics. J. Phys. C Solid State Phys..

[18] Aguado F., Rodríguez F., Hirai S., Walsh J.N., Lennie A., Redfern S.A.T. (2008). High-Pressure Behaviour of KMF_3_ Perovskites. High Press. Res..

[19] Tyagi N., Senthil Kumar P., Nagarajan R. (2010). Room Temperature Optical Absorption and Intrinsic Photoluminescence in KZnF_3_. Chem. Phys. Lett..

[20] García-Fernández P., Trueba A., García-Cueto B., Aramburu J.A., Barriuso M.T., Moreno M. (2011). Impurities Bound to Vacancies in Insulators: Electronic Relaxation and Physical Properties of the Cr^3+^ -VM Model Center in KMF_3_ (M=Mg, Zn). Phys. Rev. B.

[21] Salaün S., Rousseau M. (1995). Determination of the Fluorine-Fluorine Potential in Fluoroperovskites and Prediction of Phonon Dispersion Curves. Phys. Rev. B.

[22] Seddik T., Khenata R., Merabiha O., Bouhemadou A., Bin-Omran S., Rached D. (2012). Elastic, Electronic and Thermodynamic Properties of Fluoro-Perovskite KZnF_3_ via First-Principles Calculations. Appl. Phys. A.

[23] Maslen E.N., Spadaccini N., Ito T., Marumo F., Tanaka K., Satow Y. (1993). A Synchrotron Radiation Study of Potassium Zinc Fluoride Perovskite. Acta Crystallogr. B.

[24] Gesland J.Y., Binois M., Nouet J. (1972). Constantes Elastiques de La Fluoperovskite KZnF_3_. CR Acad. Sci. Ser. B.

[25] Brik M.G., Kumar G.A., Sardar D.K. (2012). Ab Initio, Crystal Field and Experimental Spectroscopic Studies of Pure and Ni^2+^-Doped KZnF_3_ Crystals. Mater. Chem. Phys..

[26] Qureshi M.W., Ma X., Tang G., Paudel R. (2021). Ab Initio Predictions of Structure and Physical Properties of the Zr_2_GaC and Hf_2_GaC MAX Phases under Pressure. Sci. Rep..

[27] Tripathi M.N., Saha A., Singh S. (2019). Structural, Elastic, Electronic and Optical Properties of Lead-Free Halide Double Perovskite Cs_2_AgBiX_6_ (X = Cl, Br, and I). Mater. Res. Express.

[28] Glazer A.M. (2002). WINOPTACT: A Computer Program to Calculate Optical Rotatory Power and Refractive Indices from Crystal Structure Data. J. Appl. Crystallogr..

[29] Hossain A., Alharbi H.F., Hasan M.M., Selvanathan V., Islam M.A., Homyra S., Hasan A.K.M., Haque M.M., Shahiduzzaman M., Uddin M.M. (2025). First-Principles Investigation of Electronic, Optical, and Thermal Properties of Zintl Phase MgX_2_N_2_ (X = Be, Ca, Sr) for Optoelectronic Applications: A DFT Study. Comput. Condens. Matter.

[30] Hossain A., Ali M.A., Uddin M.M., Naqib S.H., Hossain M.M. (2024). Theoretical Studies on Phase Stability, Electronic, Optical, Mechanical and Thermal Properties of Chalcopyrite Semiconductors HgXN2 (X=Si, Ge and Sn): A Comprehensive DFT Analysis. Mater. Sci. Semicond. Process..

[31] Khandy S.A., Gupta D.C. (2016). Structural, Elastic and Thermo-Electronic Properties of Paramagnetic Perovskite PbTaO_3_. RSC Adv..

[32] Akter H., Hossain M.M., Uddin M.M., Naqib S.H., Ali M.A. (2024). Effects of S Substitution on the Structural, Optoelectronic, and Thermomechanical Properties of KTaO_3_ through Density Functional Theory. J. Phys. Chem. Solids.

[33] Pugh S.F. (1954). XCII. Relations between the Elastic Moduli and the Plastic Properties of Polycrystalline Pure Metals. Lond. Edinb. Dublin Philos. Mag. J. Sci..

[34] Greaves G.N., Greer A.L., Lakes R.S., Rouxel T. (2011). Poisson’s Ratio and Modern Materials. Nat. Mater..

[35] Mayer B., Anton H., Bott E., Methfessel M., Sticht J., Harris J., Schmidt P.C. (2003). Ab-Initio Calculation of the Elastic Constants and Thermal Expansion Coefficients of Laves Phases. Intermetallics.

[36] Anderson O.L. (1963). A Simplified Method for Calculating the Debye Temperature from Elastic Constants. J. Phys. Chem. Solids.

[37] Screiber E., Anderson O.L., Soga N. (1973). Elastic Constants and Their Measurements.

[38] Wu X., Vanderbilt D., Hamann D.R. (2005). Systematic Treatment of Displacements, Strains, and Electric Fields in Density-Functional Perturbation Theory. Phys. Rev. B.

[39] Morelli D.T., Slack G.A., Shindé S.L., Goela J.S. (2006). High Lattice Thermal Conductivity Solids. High Thermal Conductivity Materials.

[40] Anderson O.L. (1959). The Debye Temperature of Vitreous Silica. J. Phys. Chem. Solids.

[41] Hossain A., Hossain M.M., Akter H., Uddin M.M., Ali M.A., Naqib S.H. (2025). Ultralow Lattice Thermal Conductivity with an Outstanding Figure of Merit of Predicted Zintl Phases: XIn_2_C_2_ (X = Sr, Ba). ACS Appl. Energy Mater..

[42] Ridou C., Rousseau M., Pernot B., Bouillot J. (1986). High-Temperature Mean Square Ionic Displacements in KZnF_3_. J. Phys. C Solid State Phys..

[43] Amudhavalli A., Rajeswarapalanichamy R., Iyakutti K., Kushwaha A.K. (2018). First Principles Study of Structural and Optoelectronic Properties of Li Based Half Heusler Alloys. Comput. Condens. Matter.

[44] Lal M., Kapila S. (2017). Structural, Electronic, Optical and Mechanical Properties of CsCaCl_3_ and KCdF_3_ Cubic Perovskites. Int. J. Mater. Sci..

[45] Giannozzi P., Andreussi O., Brumme T., Bunau O., Nardelli M.B., Calandra M., Car R., Cavazzoni C., Ceresoli D., Cococcioni M. (2017). Advanced Capabilities for Materials Modelling with Quantum ESPRESSO. J. Phys. Condens. Matter.

[46] Blöchl P.E. (1994). Projector Augmented-Wave Method. Phys. Rev. B.

[47] Perdew J.P., Burke K., Ernzerhof M. (1997). Generalized Gradient Approximation Made Simple. [Phys. Rev. Lett. 77, 3865 (1996)]. Phys. Rev. Lett..

[48] Krukau A.V., Vydrov O.A., Izmaylov A.F., Scuseria G.E. (2006). Influence of the Exchange Screening Parameter on the Performance of Screened Hybrid Functionals. J. Chem. Phys..

[49] Corso A.D. (2023). Thermo Pw Driver v.1.7.0. https://dalcorso.github.io/thermo_pw/.

[50] Nielsen O.H., Martin R.M. (1983). First-Principles Calculation of Stress. Phys. Rev. Lett..

[51] Togo A., Chaput L., Tadano T., Tanaka I. (2023). Implementation Strategies in Phonopy and Phono3py. J. Phys. Condens. Matter.

[52] Togo A. (2023). First-Principles Phonon Calculations with Phonopy and Phono3py. J. Phys. Soc. Jpn..

[53] Baroni S., de Gironcoli S., Dal Corso A., Giannozzi P. (2001). Phonons and Related Crystal Properties from Density-Functional Perturbation Theory. Rev. Mod. Phys..

